# Mesoscience in Hollow Multi‐Shelled Structures

**DOI:** 10.1002/advs.202305408

**Published:** 2023-11-30

**Authors:** Yanze Wei, Decai Zhao, Dan Wang

**Affiliations:** ^1^ State Key Laboratory of Biochemical Engineering, Institute of Process Engineering Chinese Academy of Sciences Beijing 100190 P. R. China; ^2^ Key Laboratory of Biopharmaceutical Preparation and Delivery Chinese Academy of Sciences Beijing 100190 P. R. China; ^3^ University of Chinese Academy of Sciences Beijing 100049 P. R. China

**Keywords:** compromise in competition principle, hollow multi‐shelled structure, mesoscale, mesoscience, multi‐functional material

## Abstract

The prevalence of mesoscale complexity in materials science underscores the significance of the compromise in competition principle, which gives rise to the emergence of mesoscience. This principle offers valuable insights into understanding the formation process, characteristics, and performance of complex material systems, ultimately guiding the future design of such intricate materials. Hollow multi‐shelled structures (HoMS) represent a groundbreaking multifunctional structural system that encompasses several spatial regimes. A plethora of mesoscale cases within HoMS present remarkable opportunities for exploring, understanding, and utilizing mesoscience, varying from the formation process of HoMS, to the mesoscale structural parameters, and finally the distinctive mass/energy transfer behaviors exhibited by HoMS. The compromise in competition between the diffusion and reaction contributes to the successful formation of multi‐shells of HoMS, allowing for precise regulation of the structural parameters by dynamically varying the interplay between two dominances. Moreover, the distinct roles played by the shells and cavities within HoMS significantly influence the energy/mass transfer processes with the unique temporal‐spatial resolution, providing guidance for customizing the application performance. Hopefully, the empirical and theoretical anatomy of HoMS following mesoscience would fuel new discoveries within this promising and complex multifunctional material system.

## Graphical abstract

Through the lens of mesoscience, the review conducts a comprehensive analysis of the mesoscale issues involved in the formation process of HoMS, the structural parameters of HoMS, and performance in energy/mass transfer, where the compromise in competition principle roles. By establishing the crucial connection between process and appearance, mesoscience provides a new perspective on the underlying mechanisms within HoMS chemistry.

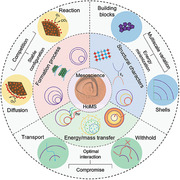

## 1 Introduction

**Figure 1:**
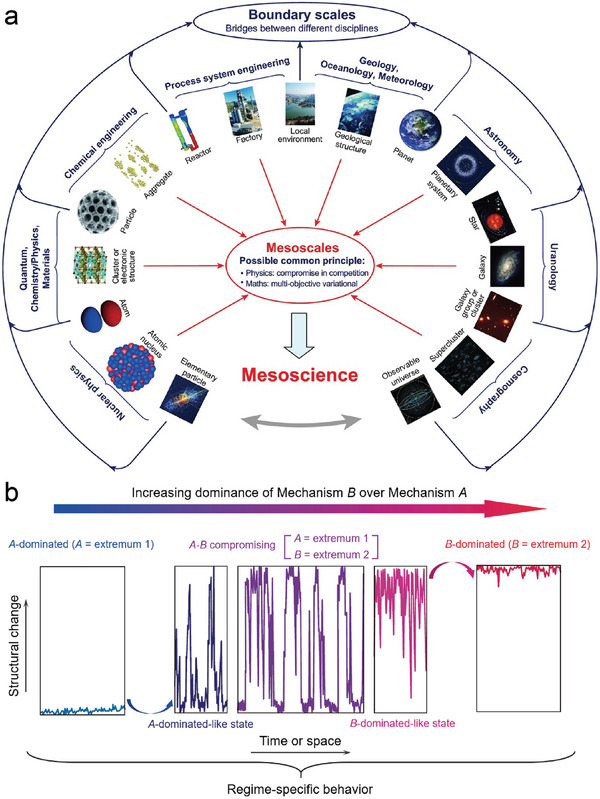
a) A unified theory of mesoscience will encompass all mesoscale phenomena: this represents a common challenge for the whole spectrum of science and technology.^[^ ^1^ ^]^ Reproduced with permission. Copyright 2018, Oxford University Press. b) Three regimes occur successively with changing the relative dominance of Mechanism B over Mechanism A. The principles and rules of compromise in competition can be explained as follows: 1) A‐dominated: When A is close to the critical value (A = extremum 1), A takes control and suppresses B (B = extremum 2). As a result, the system structure is solely determined by A, and the impact of B on the system structure could be disregarded. 2) A‐B compromise in competition: As the dominance of B increases over A, the dominance of A diminishes. When the critical point is reached, A must compromise with B and loses its dominance. The system is no longer fully dominated by A, but rather by the relative dominance between these two mechanisms. 3) B‐dominated: When B reaches the critical value, the system structure is entirely defined by B, and the influence of A is disregarded.^[^ ^12^ ^]^ Reproduced with permission. Copyright 2016, Elsevier.

One puzzling cloud over modern scientific society, which obscures the beauty and clearness of delicate complex systems, is the limited understanding of the boundary and the internal regimes scales between different spatial or temporal dimensions.^[^ ^1^, ^2^, ^3^, ^4^, ^5^ ^]^ Revealing these intermediate scales is highly desired as it helps bridge the gap between different scales and harmonize the heterogeneity in systems with high spatiotemporal diversity, which gives birth to a transdisciplinary field: mesoscience.^[^ ^6^ ^]^ Derived from ancient Greek, the term “meso” in “mesoscience” pertains to the middle or intermediate, symbolizing the distinct phenomenon found at the mesoscale between two boundary scales.^[^ ^7^ ^]^ Mesoscale refers to the intermediate scale between micro and macro, where a characteristic structure called meso‐structure exists. This meso‐structure exhibits dynamic heterogeneity in space and time, which is crucial for the system's performance. Parameters at the mesoscale are necessary to connect the mechanisms at the element scale to the system's behavior. The goal of mesoscience is to establish a general principle that can effectively bridge different disciplines at various levels (**Figure** **1**a). The concept of mesoscience is surprisingly instructive in materials science, as it would effectively discover the complexity of collective behaviors in a wide range of material systems.^[^ ^8^, ^9^, ^10^, ^11^ ^]^ Notably, excellent modeling has been developed by the Li group, in which the system performance is determined by two mechanisms, A (extremum 1) and B (extremum 2), as depicted in Figure 1b.^[^ ^12^ ^]^ The field of mesoscience is guided by the “energy minimization multi‐scale (EMMS) principle,” which highlights the compromise in competition between different dominant mechanisms.^[^ ^13^ ^]^ With the established physical and mathematical expressions, mesoscience provides valuable insights into the synthetic methods,^[^ ^14^, ^15^, ^16^, ^17^, ^18^, ^19^ ^]^ characteristics,^[^ ^20^, ^21^, ^22^, ^23^ ^]^ and performance^[^ ^24^, ^25^, ^26^, ^27^, ^28^ ^]^ of complex material systems, yielding strikingly architectures, phenomena, and functionalities. However, the continued advancement of mesoscience in materials science calls for a multi‐scale platform that incorporates temporal‐spatial mesoscales, which would successfully expand the applicability of mesoscience in discovering precise regulation strategies and uncovering new features.^[^ ^1^ ^]^

Fortunately, with fruitful developments, the rising of hollow multi‐shelled structures (HoMS) opens up new opportunities for exploring mesoscience concepts in both spatial and temporal regimes.^[^ ^29^, ^30^, ^31^, ^32^ ^]^ Owing to the distinct characteristics at different levels within this 3D hollow structure, several meso‐regimes are effectively segregated by well‐defined boundaries ranging from building blocks, shells, and the ordered multiple shells in HoMS.^[^ ^33^ ^]^ The formation process of HoMS involves the initial arrangement of nanoparticles into individual shells, followed by the assembly of these shells into a hierarchical structure.^[^ ^34^, ^35^, ^36^ ^]^ Notably, the rate of shell formation and template removal engage in a competitive process, demonstrating the principle of compromise within physical space and temporal sequence.^[^ ^29^ ^]^ By analyzing the multiple synergistic and competitive relations between adjacent spatial scales, the precise modifying of structural parameters of HoMS could be realized by matching reactions at different time regimes. Additionally, regime‐specific behaviors occur within the shell structures and the cavities, which govern the mass/energy transfer process in HoMS and provide a bunch of mesoscience cases.^[^ ^37^ ^]^ Accordingly, the transdisciplinary nature of HoMS offers an exceptional platform for analyzing the intricate mesoscience present in complex material systems, thereby unlocking its potential for solving complex problems facing practical applications.^[^ ^38^ ^]^

In this review, through the lens of mesoscience, we meticulously examine the intricate HoMS system. Our investigation commences by delving into the concept of compromise‐in‐competition within the formation process of HoMS. Subsequently, we conduct a thorough analysis of the shell structure inherent in HoMS, and finally, we explore the diverse application scenarios where HoMS can be effectively utilized. By precisely defining the boundaries of the meso‐regime, we gain valuable insights into the dominant mechanisms that contribute to the analysis of mesoscale phenomena arising from the underlying compromise‐in‐competition principle. In essence, the formation of HoMS stems from the intricate interplay between diffusion and reaction, exerting a profound influence on the crystal and geometric structure of both building blocks and shell structures. Spatially, the building blocks and shell structures play a crucial role in establishing two distinct boundary scales – one for the individual elements and another for the overall system. These boundary scales serve as the essential connecting link between the formation and application of HoMS. In terms of temporal‐spatial dynamics, the shells and cavities within HoMS govern two distinct mechanisms for energy and mass transfer, thereby offering novel avenues for tailoring the application performance of HoMS. Studying HoMS from the vista of mesoscience not only opens up new sights to discover the unknown intrinsic properties of HoMS but also effectively reflects common physical laws in the mesoscales. Most importantly, by leveraging the theoretical framework of mesoscience, the establishment of a physical and mathematical understanding of the structure and behavior of HoMS holds immense potential for advancing the field. This knowledge could enable the development of customized fabrication techniques, allowing for the creation of HoMS with precisely designed functions and desired performance characteristics. Such advancements would open up new possibilities for their application in a wide range of industries and domains.

## 2 Relationship Between Mesoscience and HoMS

In the realm of modern scientific study, the concept of mesoscience is prominently observed within the domains of physics, chemistry, biology, geology, and ecology.^[^ ^39^, ^40^, ^41^, ^42^, ^43^ ^]^ These quintessential fields, which encompass meso‐related phenomena, exhibit a remarkable degree of complexity and diversity due to the presence of collective effects.^[^ ^44^, ^45^, ^46^, ^47^, ^48^, ^49^ ^]^ Consequently, an intermediate level, mesoscale, emerges between the elemental scale and the system scale, giving rise to rich case studies of interactions and structures.^[^ ^50^, ^51^ ^]^ However, confusion in related research often arises from overlooking the differences between mesoscale regimes and their limiting cases.^[^ ^52^, ^53^, ^54^ ^]^ To advance the field of mesoscience, HoMS offers valuable case studies to explore hidden mesoscale phenomena from the EMMS or compromise‐in‐competition principle.^[^ ^55^ ^]^ The transdisciplinary feature of HoMS provides an excellent platform to analyze the mesoscales within two or more dominances, thus delivering some common rules in the mesoscience concept to the scientific community.

In the field of HoMS, mesoscience offers a fresh perspective that emphasizes the process and intermediate scales within HoMS. This viewpoint provides a deeper understanding derived from the compromise and competition relationships between these scales. Specifically, three main aspects are provided by mesoscience:

1. By analyzing the relationship between diffusion and reaction in the synthetic method of HoMS, the physical and mathematical representations of mesoscience can serve as theoretical supports for numerical explaining the formation process of HoMS and providing guidance for precise regulation of structural parameters.
2. By understanding the synergistic and competitive relationship between building blocks and shell structures as individual elements and the overall system, the influence of individual variations on the spatial configuration and physicochemical property changes of the system can be comprehended.
3. By clarifying the distinct mechanisms of shell structures and cavity structures in energy/mass transfer processes, the maximum transfer efficiency and smart responses could be achieved with the optimal spatiotemporal distribution of shell structures for balancing the withholding and transport of energy/mass.

**Figure 2:**
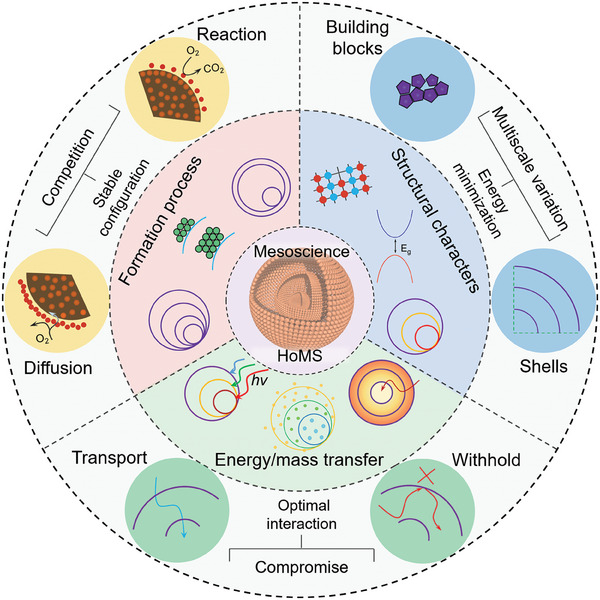
Schematic illustration of the relationship between mesoscience and HoMS. The formation of HoMS is a result of the intricate interplay between diffusion and reaction, which has a profound impact on the number of shells, the thickness of shells, and the inter‐shell spacing. The building blocks and shell structures of HoMS create two distinct spatial boundaries. This multiscale variation allows for the system to minimize its energy, thereby significantly affecting the electronic structure, geometry, and physicochemical properties of HoMS. For energy/mass transfer in HoMS, the shells and cavities rule differently in withholding and transporting, respectively, thus creating a meso‐regime. By achieving optimal interaction after the compromise, this meso‐regime enables the achievement of enrichment effects, controlled release, and sequential responses.

Overall, a comprehensive analysis of the multiple synergistic and competitive relationships among diverse temporal and spatial boundary conditions offers a scientific and dialectical framework for comprehending HoMS, which paves the way for accurate synthesis and regulation of shell structures to cater to specific application scenarios. This contribution has the potential to bridge knowledge gaps in modern materials science and chemistry. Furthermore, investigating various mesoscale aspects of HoMSs can uncover common principles in mesoscience, offering valuable insights for the design of novel complex material systems (**Figure** **2**).

## 3 Mesoscience in the Formation of HoMS

The successful fabrication and precise regulation of HoMS represents a remarkable achievement in synthetic chemistry, showcasing the application of mesoscience concepts in the design of multivariate and multiscale mesostructures.^[^ ^56^ ^]^ Experimentally, mesoscience operates on the principle of compromise amidst competition, which arises from the multifaceted variations observed mathematically.^[^ ^57^ ^]^ Consequently, HoMS can be seen as a mesoscale assembly of nanoparticles that adheres to a common mesoscience law, namely the equilibrium between shell formation and template removal rates.^[^ ^58^ ^]^ Furthermore, the mathematical expression exposes the two prevailing mechanisms: the reaction process and the diffusion process. To gain a comprehensive understanding of the formation process of HoMS, this section extensively reviews both the experimental data that capture the physical characteristics and the theoretical analysis that encompasses the underlying mathematical essence.

### 3.1 Experimental Observations on the Formation of HoMS

**Figure 3:**
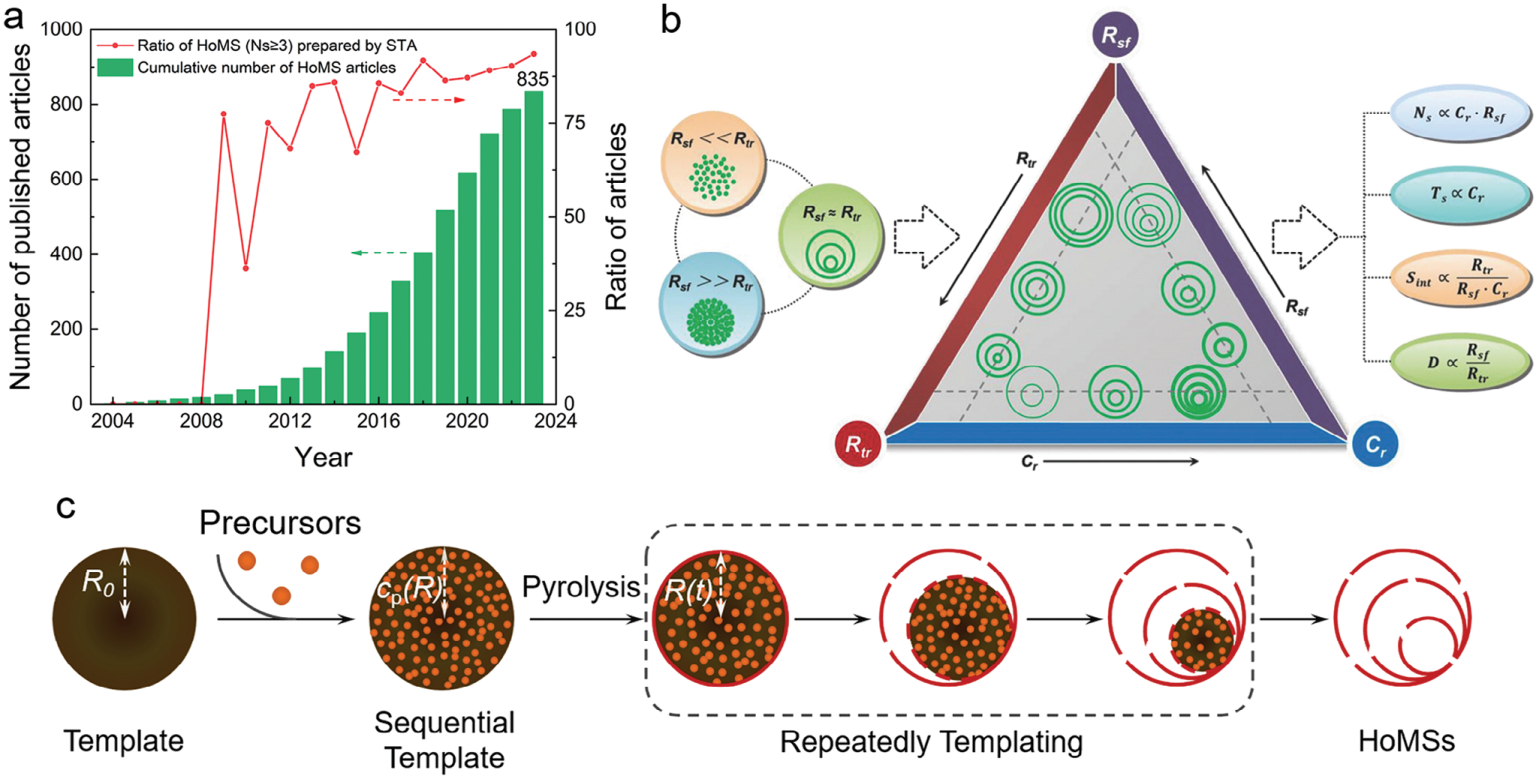
a) Statistical number of published papers about HoMS prepared by STA and other methods, and the yearly changed ratio of HoMS (shell number ≥3) prepared via STA till 2023. b) Illustration of the inter‐correlations among the key parameters of STA and their impacts on the formation of HoMSs. Reproduced with permission.^[^ ^59^ ^]^ Copyright 2018, Wiley‐VCH. c) Schematic illustration depicting sequential templates throughout the STA, with the diameter of shells set to be equal to the diameter of templates at the moment of separation. Reproduced with permission.^[^ ^60^ ^]^ Copyright 2023, Wiley‐VCH.

The attention and research on HoMS materials began in 2004. Despite their attractive and unique physicochemical properties and potential applications, the complex synthesis steps have hindered their further development. To break the constrains for the synthesis of HoMS, Wang's group and collaborators established a general approach using sequential templates in 2009, namely the sequential templating approach (STA). Prof. Rose Amal, a Fellow of the Australian Academy of Science, believes that the development of STA as a pioneering achievement, “The breakthrough in synthesis methodology was rapidly embraced by scientists worldwide, with various groups adopting the approach where HoMSs were identified as promising materials for many applications”.^[^ ^61^ ^]^ Since then, the synthesis of HoMS has been flourished, leading to the publication of 835 articles on the subject. Among them, ≈84% of the total reported HoMS with three or more shells have been successfully fabricated using the STA method (**Figure** **3**a).^[^ ^62^ ^]^ As a result, this review primarily focuses on the experimental observations pertaining to the formation of HoMS through the STA method.

In a general view, the fabrication process of HoMS begins with the preparation of sequential templates. The properties of these templates, such as their composition,^[^ ^63^, ^64^, ^65^, ^66^, ^67^ ^]^ surface functional group,^[^ ^68^, ^69^ ^]^ and charge distribution,^[^ ^70^ ^]^ have a significant impact on the distribution of precursors within them. The subsequent step involves the removal of these templates, which are influenced by both thermodynamic and kinetic factors. During this template removal process, the formation of the shell structure occurs through the dehydration, aggregation, and crystallization of precursors. This process also leads to the repeated formation and detachment of shells, ultimately resulting in the creation of multi‐shell structures and corresponding cavities in HoMS. Thanks to the numerous fabrication attempts and regulatory efforts made in the past decade, we now have sufficient data to construct an experimental understanding of the formation of HoMS. The collective effects of various elements, such as the “shell formation rate (*R*_sf_),” the “template removal rate (*R*_tr_),” and the “precursor amount (*C*_r_),” provide several regimes that can be used to modulate the formation of HoMS.^[^ ^59^ ^]^ In light of this, a qualitative framework analysis has been proposed by Mao et al to explain the formation process. Coincidently, as depicted in Figure 3b, the formation of HoMS exhibits several distinct meso‐regimes that effectively illustrate the underlying mesoscience governing their formation and the regulation of shell structures.

Primarily, the formation of HoMS can be seen as the result of a compromise in the competition of *R*_sf_ and *R*_tr_. A faster calcination rate accelerates *R*_tr_ and the aggregation of precursors, thereby promoting the assembly of as‐formed building blocks, which ultimately leads to the production of fragile shell debris. Conversely, a slower calcination rate inhibits the consumption of templates and instead promotes the crystal growth of building blocks, resulting in the formation of bulk materials. Thus, nanoparticle‐debris and bulk materials are two extremal results, which construct the two regimes that dominate the shell formation process. The relationship between *R*_sf_ and *R*_tr_ is described as follows:

(1) $$R_{\mathrm{sf}}∼R_{\mathrm{tr}}$$

Additionally, the precise control of shell structure parameters, such as the shell number (*N*_s_), shell thickness, inter‐shell spacing, and shell diameter, also follows the “compromise in competition” mesoscience principle. Among these parameters, the regulation of *N*_s_ is the most extensively studied due to the multi‐shell feature of the HoMS systems. The process involves the dehydration and crystallization of the precursors, as well as the packing of the resulting nanoparticles and their movement with the templates (Figure 3c).^[^ ^60^ ^]^ These processes are in competition with each other, resulting in variations in the packing density of the shells and the rate at which precursors are consumed. When a specific *C*_r_ is used, the close interaction between nanoparticles and templates can dominate the packing, resulting in closely‐packed shells and decreased *N*_s_.^[^ ^71^ ^]^ On the other hand, excessive detachment can lead to incomplete shells that separate from the template one after another, resulting in fragile shell structures.^[^ ^72^, ^73^ ^]^

**Figure 4:**
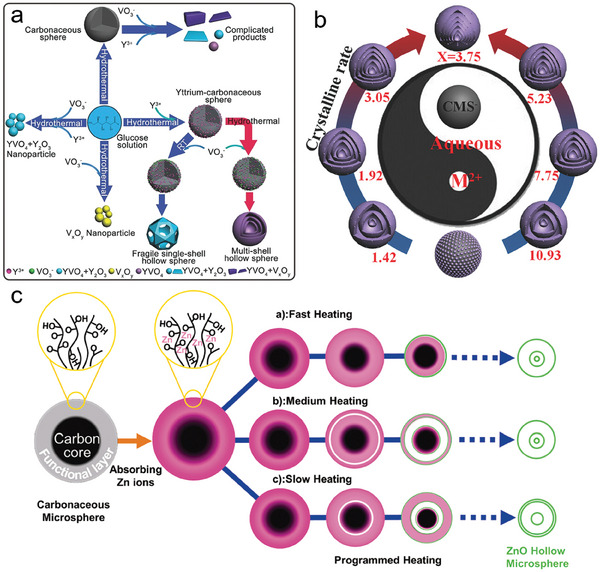
a) Illustration of the formation exploration for YVO_4_ HoMS through various approaches.^[^ ^73^ ^]^ Reproduced with permission. Copyright 2017, Wiley‐VCH. b) Illustration of the formation of (Co_1−x_Mn_x_)(Co_1−y_Mn_y_)_2_O_4_ HoMS with different *N*_*s*_ by finely controlling the molar ratio of Co/Mn using sequential templating method (M^2+^: Co^2+^ and Mn^2+^; X: the molar ratio of Co/Mn).^[^ ^73^ ^]^ Reproduced with permission. Copyright 2017, Wiley‐VCH. c) Illustration of the formation of multishelled ZnO hollow microspheres through different heating processes.^[^ ^74^ ^]^ Reproduced with permission. Copyright 2012, Wiley‐VCH.

For instance, in the case of a shell composition with a slow crystallization rate, such as bimetallic oxide, the process of shell separation is primarily influenced by the interaction between the precursor and templates.^[^ ^75^ ^]^ The experimental results demonstrate that by sintering the sequential templates at a programmed heating rate of 0.5, 1, and 2 °C min^−1^, it is possible to obtain single‐, double‐, and triple‐shelled YVO_4_ HoMS, respectively.^[^ ^73^ ^]^ In this case, the increased heating rate is adopted to reduce the dominance of the interaction between precursor and template, thus accelerating the *R*_sf_ to keep pace with the *R*_tr_. It is worth noting that in this particular case, the use of yttrium‐composited CMSs with adsorbed VO_3_^−^ ions is strongly recommended. This observation indicates that increased interaction among the precursors would compete with the interaction between the precursor and templates. As a result, the formation of multi‐shells can be seen as a compromise between these two dominant factors (**Figure** **4**a). Another example of regulating *N*_s_ involves tuning the *R*_sf_ through compositional engineering by altering the molar ratio of metal cations in the sequential templates. As illustrated in Figure 4b, in the case of Co ions with a catalytic combustion effect, where the *R*_tr_ plays a dominant role in the formation of Co‐based HoMS. By doping Mn ions into the matrix, the dehydration and crystallization rate of spinel Co_3_O_4_ can be modified, thereby changing the *R*_sf_. Through the addition of different amounts of Mn precursors to compete with the *R*_tr_, septuple‐shelled (Co_2/3_Mn_1/3_)(Co_5/6_Mn_1/6_)_2_O_4_ HoMS were successfully produced.^[^ ^76^ ^]^ Consequently, the precise manipulation of *N*_*s*_ serves as an illustrative example of modifying the multi‐variational criterion to alter the operational regime for the formation of HoMS. By engaging in a dynamic interplay of competition and compromise, the dominant mechanisms can be modified, leading to the creation of novel structures.

For shell thickness, although it is positively correlated with *C*_r_ and negatively with *R*_tr_, the essence is also dominated by the mesoscience regarding the interactions between nanoparticles and templates. The solid sphere and nanoparticle debris are two typical products resulting from the two dominances. Hence, the calcination procedure is crucial for obtaining desired shell thickness.^[^ ^77^ ^]^ The real condition for tuning the shell thickness is more complex, as the “precursor distribution” plays another important role. With a certain composition, the interaction between nanoparticles varies with the density of the surrounding precursors. For example, by regulating the size of tungsten precursors via solvent engineering, Zhang et al optimized the radial distribution of tungsten within the CMSs, thus successfully realizing the accurate control of the shell thickness of as‐formed WO_3_ HoMS in the range of 35–90 nm.^[^ ^78^ ^]^

Considering the distribution of precursor distribution, it is observed that the inter‐shell spacing exhibits a positive correlation with *R*_tr_, while displaying a negative correlation with *R*_sf_ and *C*_r_. In the case of sequential templates with surface accumulated precursor at the same *C*_r_, the inter‐shell spacing demonstrates two extremes: the adjacent multi‐shells and the solid spheres. Consequently, it is evident that the inter‐shell spacing is greatly influenced by the calcination procedure. Dong et al. have systematically investigated the influence of heating rate on inter‐shell spacing.^[^ ^74^ ^]^ As shown in Figure 4c, a fast heating rate leads to the formation of a thin exterior shell, as fewer Zn ions are present within the exterior shells due to rapid template removal. Conversely, a slow heating rate results in a higher concentration of Zn ions within the exterior shells, leading to the formation of a closed double shell in the resulting ZnO HoMS. Additionally, the role of precursor distribution with similar *C*_r_ is also validated by experiments. Pretreating the CMSs with alkali facilitates the generation of an increased concentration of negatively charged hydroxyl surface groups on the CMSs. This, in turn, enhances the absorption of Sn^4+^ within the CMSs, ultimately resulting in the formation of SnO_2_ HoMS with a closed double shell by changing the precursor distribution.^[^ ^79^ ^]^

### 3.2 Theoretically Analysis of the Formation of HoMS

The abundant experimental results and ex‐situ observations of the formation and structural regulation of HoMS have provided valuable insights into the essential role played by several dominant mechanisms, which embody the essence of mesoscience.^[^ ^76^ ^]^ However, despite these intriguing experimental observations, the precise contribution of mesoscale science in the development of HoMS remains unexplained. To embrace the challenge and overcome the drawback in understanding the formation process of HoMS, the theoretical analysis may help to unravel the black box.^[^ ^80^, ^81^ ^]^ Most recently, Wang's group established an effective numerical technique to provide mathematical expressions for the formation process of HoMS when utilizing STA.^[^ ^60^ ^]^ By simplifying the dehydration and crystallization reactions of the precursors, they identified the oxidation reaction of templates and the diffusion of oxygen to the surface of templates as the two key dominance mechanisms in STA that dictate the formation of shell structures.^[^ ^82^, ^83^ ^]^ According to the numerical model, the surface precursor concentration as a function of the radius of the template changes could be described as:

(2) $$\frac{dR}{dt}=-\frac{V_{m}}{\frac{h}{\left(1-x\right)Dc_{E}}+\frac{1}{A\cdot e^{-\frac{E_{a}}{k_{B}T}}\cdot c_{E}}}$$

where A is the pre‐exponential factor in the Arrhenius equation, *E*_a_ is the activation energy, *V*_m_ is the molar volume of CMSs counted by carbon element, *h* is the thickness of the shell, *x* is the coverage ratio of shell, *D* is the diffusion factor of oxygen, and *c*_E_ is the oxygen concentration in the environment. In Equation (2), $\frac{h}{\left(1-x\right)Dc_{E}}$ reflects the influence of O_2_ diffusion, while $\frac{1}{A\cdot e^{-\frac{E_{a}}{k_{B}T}}\cdot c_{E}}$ reflects the influence of reaction rate. Therefore, the real‐time *R_tr_* is influenced by both the diffusion and reaction processes. It is worth noting that the diffusion process is particularly sensitive to the surface properties of templates, as any alterations in *h* and *x* would collectively impede the access of O_2_ to the template surface.

**Figure 5:**
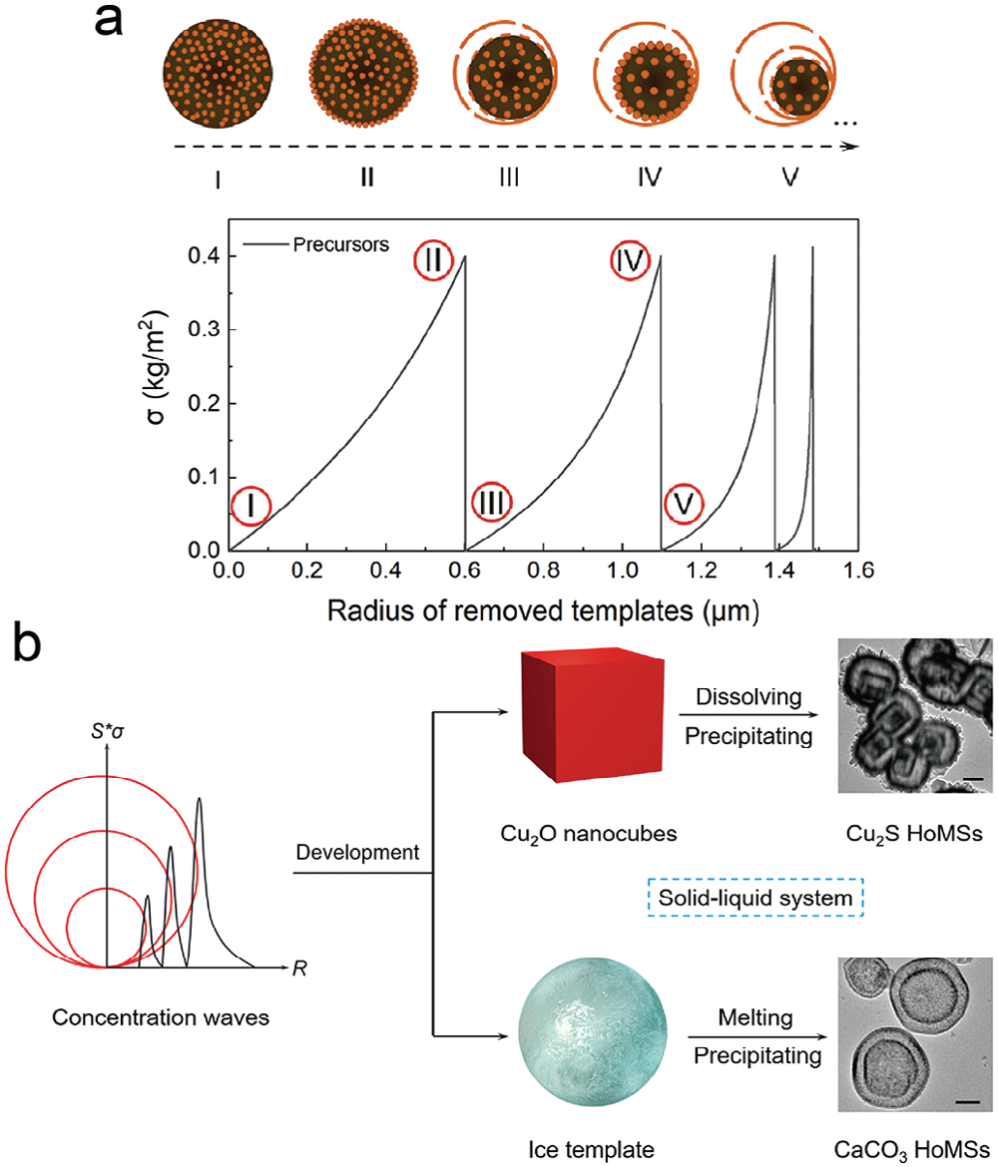
a) Variation of the σ during the template removal process and the schematic illustrations of labeled states. b) Experiment results of the conversion of Cu_2_O templates to Cu_2_S HoMS, and c) CO_3_^2−^ enriched ice templates to CaCO_3_ HoMS in solution systems (scale bar: 200 nm).^[^ ^60^ ^]^ Reproduced with permission. Copyright 2023, Wiley‐VCH.

The identification of these two dominant factors in the shell formation process strongly indicates the presence of a meso‐regime that experiences the principle of compromise in competition. Based on the simulation results, the removal of the template leads to the accumulation of precursors on the surface, which subsequently hinders the diffusion of oxygen and decelerates the oxidation rate of the template surface. However, once the concentration of precursors reaches a point where they fully cover the surface of templates, the reaction becomes greatly hindered. This hindrance leads to an abrupt decrease in the reaction rate as the precursors detach from the template. The detachment process allows oxygen to regain unrestricted access to the template surface. The interplay between diffusion dominance and reaction dominance gives rise to a mesoscale regime, characterized by compromising of diffusion and reaction. A notable manifestation of this regime‐specific behavior is the gradual formation of shell structures due to the accumulation of precursors on the surface of templates. Numerical simulation techniques not only offer valuable insights into the underlying physical mechanisms of the synthetic strategy but also uncover the mesoscience governing the formation process of HoMS. Through the aid of a more intuitive spectrum, the results of these simulations reveal that the surface concentration of precursors (*σ*) exhibits a periodic variation akin to waves during the template removal process (**Figure** **5**a). More importantly, the research team emphasizes that the key aspect of the formation of HoMS lies in generating concentration waves within the meso‐regime. Building on this theoretical insight, the authors successfully fabricate Cu_2_S HoMS and CaCO_3_ HoMS in solution systems with low temperatures using Cu_2_O templates and CO_3_^2−^‐enriched ice templates, respectively (Figure 5b). Overall, this milestone study provides a deep glimpse into the significant role of mesosciences in synthesizing and structurally regulating complex mesoscale materials. It also confirms the impact of the compromise in competition principle on the structure during the material synthesis process.

In this section, we delve into the intricate relationship between *R*_sf_ and *R*_tr_, guided by the principles of mesoscience, which highlights the interplay between diffusion and reaction during the formation process of HoMS. The experimental results demonstrate the fabrication and regulation strategy of shell structures by finely adjusting the balance between *R*_sf_, *R*_tr_, and *C*_r_ at various mesoscales. The physical and mathematical representations obtained through mesoscience provide a solid theoretical framework for numerically understanding the intricate formation process of HoMS. Furthermore, by carefully controlling the competition between these two mechanisms, the precise regulation of shell parameters could be achieved, enabling meticulous control over the synthesis of HoMS.

## 4 Mesoscience in the Structure of HoMS

**Figure 6:**
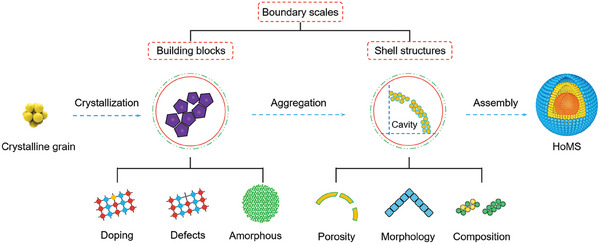
Illustration of mesoscience in the structure of HoMS.

After exploring and identifying the mesoscience involved in the formation of HoMS, our investigation now focuses on examining the various mesoscale issues manifested within the structure of HoMS. In terms of spatial complexity, the mesostructured HoMS can be primarily divided into two boundary scales: building blocks and shell structures. The arrangement of crystalline grains through crystallization plays a crucial role in determining the composition and properties of building blocks, which in turn impact the formation of shell structures. Similarly, the structural parameters and composition of shell structures are closely intertwined with the building blocks. As a result, these two boundary scales act as bridges between various disciplines or regimes, enabling a comprehensive comprehension of mesoscience within the system. It is crucial to emphasize that the discussion regarding mesoscience in this article must be closely linked to the unique structural characteristics of HoMS or the featured formation process, as it is within this context that the discovery of mesoscience within HoMS is situated **Figure** **6**.

### 4.1 Mesoscience in the Building Blocks of HoMS

The building block serves as the fundamental unit for constructing shell structures, effectively bridging the gap between the spatially distinct quantum‐sized scale and the nano‐sized scale.^[^ ^84^ ^]^ Crystallization of crystalline grains via solid‐state reactions remains the primary method for constructing these building blocks, mainly achieved through STA. Other methods, such as polymerization or ion exchange, can also be employed to effectively organize grains or molecules and form interconnected building blocks.^[^ ^85^, ^86^ ^]^ Various dominant mechanisms within this mesoscale realm intricately interplay with one another to forge the structural and physicochemical properties of the building blocks. This leads to the emergence of numerous mesoscale phenomena, offering ample opportunities for exploration and discovery.^[^ ^87^ ^]^ Moreover, given that the formation of HoMS through STA has been established as the manifestation of concentration waves, it is important to note that the properties of building blocks, including their electronic structure, crystal structure, and morphology, are significantly influenced by the guiding concentration waves.^[^ ^88^ ^]^ As a consequence, the multi‐objective variations in the formation of HoMS give rise to nontrivial properties in building blocks.

To date, the composition of HoMS primarily consists of metal oxides or related derivatives, such as sulfides, phosphides, or nitrides.^[^ ^35^ ^]^ The geometric structural parameters and physicochemical properties of these composites are heavily influenced by the crystal structure of the constituent building blocks.^[^ ^89^ ^]^ Therefore, the crystallization process should be given primary consideration when studying mesoscience in the building blocks of HoMS. Associated with the formation of HoMS, the crystal structure of the building blocks is intricately linked to the properties and distribution of precursors in the sequential templates. During the removal of templates, dehydration, and aggregation occur simultaneously, followed by crystallization.^[^ ^34^ ^]^ The STA method, influenced by environmental factors such as temperature and atmosphere, produces HoMS consisting of building blocks with varying sizes and morphologies, which are influenced by different crystal structures. Herein, we investigate the predominant roles of ordering and disordering in the mesoregime, which arise from the compromise and competition during crystallization. This exploration uncovers the mesoscience aspect, which revolves around the multifaceted variations in size, crystallization, and electronic states of the building blocks. Consequently, the magnetic properties, charge separation efficiencies, and surface adsorption efficiencies undergo significant alterations, resulting in modified features for electrocatalysis, photocatalysis, and electromagnetic‐wave absorption applications.

**Figure 7:**
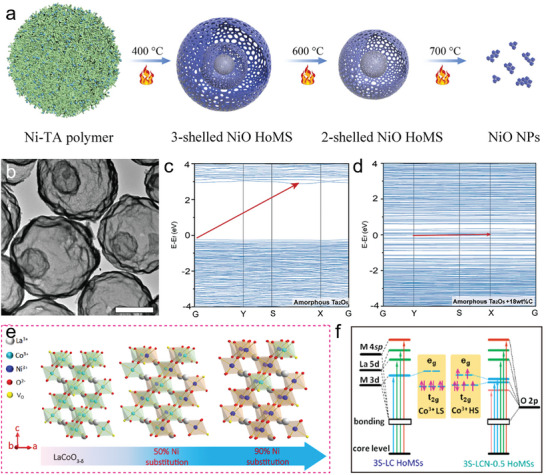
a) Schematic diagram for the formation of NiO HoMS with different shell numbers.^[^ ^90^ ^]^ Reproduced with permission. Copyright 2016, Elsevier. b) TEM image of Ta_2_O_5_/C HoMSs (scale bar, 500 nm). c) Band structures of amorphous Ta_2_O_5_ and d) amorphous Ta_2_O_5_/C composite.^[^ ^91^ ^]^ Reproduced with permission. Copyright 2022, Wiley‐VCH. e) Schematic illustration of crystal structure for LaCo_1‐x_Ni_x_O_3‐δ_ HoMSs with different Ni substitution. f) The simple model of the XAS absorption processes (The light blue, light green, and orange area correspond to the transition from the core level to the hybridization states O 2p and M 3d, O 2p and La 5d as well as O 2p and M 4sp, respectively).^[^ ^92^ ^]^ Reproduced with permission. Copyright 2020, Wiley‐VCH.

The amorphous phase‐crystallized phase transition is the most direct manifestation of ordering‐disordering at the boundary scale that consists of the building blocks. According to the governing principle in mesoscales, known as EMMS, three distinct regimes emerge in the crystallization condition of the shells: the A‐dominated regime, characterized by a crystallinity of 100% under ideal conditions; the B‐dominated regime, where the shell remains entirely amorphous; and the middle regime, where a coexistence of crystals and “non‐crystals” is observed.^[^ ^93^ ^]^ In general, the regulation of crystallinity within the meso‐regime can be classified into two categories. According to a superficial phenomenon, the size of building blocks is closely related to crystallinity. The first category primarily applies to single‐component HoMS, where the utilization of crystallinity plays a crucial role in controlling the geometry of building blocks. In turn, the variations significantly affect the structural parameters of shells. For example, by bumping the calcination temperature, the formation of NiO HoMS with different shells is the result of the EMMS principle within the mesoscale divided by amorphous Ni‐based templates and highly crystallized NiO nanoparticles, as discussed in the last section.^[^ ^90^ ^]^ The increasing crystallinity of NiO building blocks competes with the amorphous nature of the NiO precursors, which allows the changing in the morphology and porosity of NiO shells (**Figure** **7**a). Moreover, as crystallization would happen after the formation of HoMS, crystal growth by simple heat treatment gives a superficial example of the influence of crystallinity on the structural and physicochemical properties of HoMS. Upon heating the obtained NiO HoMS to a temperature of 800°C, the crystal growth process induces the inward collapse of the shells, ultimately leading to the formation of NiO nanoparticles. Consequently, the relationship between the crystallinity of building blocks and the shell structures is related via the compromise in competition within the meso‐regime.

On the other hand, for the second category, the mesoscale of building blocks within the amorphous and crystalline structures is thoroughly examined and harnessed to control the physicochemical characteristics of HoMS.^[^ ^6^ ^]^ These investigations are specifically conducted with a focus on the diverse chemical bonding present in the building blocks, allowing for precise manipulation of the desired properties. For example, when modifying the atmosphere to eliminate the Ta‐precursor loaded CMSs, the competition in the formation of C─Ta─O or Ta─O bonds significantly impacts the arrangement of the edge‐ and corner‐shared TaO_6_ octahedra and TaO_7_ pentagonal bipyramids in the quasi‐octahedral coordinated Ta_2_O_5_ crystal,^[^ ^94^ ^]^ resulting in the amorphous domain in the building blocks (Figure 7b).^[^ ^91^ ^]^ The created amorphous counterparts successfully altered the electronic structure of Ta_2_O_5_ building blocks, which converts the direct band gap of Ta_2_O_5_ into a narrow indirect band gap with enhanced response to a wide light spectrum (Figure 7c,d). Additionally, the distorted crystals are more sensitive to the photo‐induced phonons due to an amplified electron‐lattice interaction, thus empowering Ta_2_O_5_/C HoMS with exceptional photo‐thermal performances for solar‐vapor generation.

Apart from the composited shells, the introduction of element doping leads to a disruption in the crystal structure, which is influenced by two dominant crystal matrices. The resulting new lattice formed in this meso‐regime is significantly influenced by the two original crystals.^[^ ^95^ ^]^ The compromise after the competition would result in abnormal crystal parameters and, more importantly, alterations in the electronic structure. In a typical work, Wang et al. employed the flexible STA method to develop LaCo_1‐x_Ni_x_O_3‐δ_ HoMSs, allowing precise control over the metal precursor content within the templates (Figure 7e).^[^ ^92^ ^]^ By gradually replacing Co^3+^ ions in the B site with Ni^2+^ ions of alternating valence, the spin state of Co^3+^ ions is effectively adjusted from a low spin state to a high spin state, resulting in reduced charge transport resistance. This spin state evolution is confirmed by the O K‐edge X‐ray absorption spectroscopy (XAS) spectra, which show a noticeable red shift of Co 3d‐related peaks toward lower energy, indicating a decrease in crystal‐field splitting energy (10Dq). Consequently, the high‐spin state of Co leads to an increased occupancy of electrons in the e_g_ state of the Co 3d orbital within the [CoO_6_]^8−^ octahedral unit, enhancing the hybridization between Co 3d states and O 2p states (Figure 7f). Simultaneously, the substitution of Ni^2+^ ions in the building blocks introduces surface oxygen vacancies and lattice distortions in the LaCoO_3_ crystal, which further reduces the charge transport resistance within the subunits.

Additionally, at the atomic level, the uncertainty principle in doping sites originates from the coexistence of some competing mechanisms. For example, originating from different doping sites, the surface defects building blocks experience two states, which imply a crystal‐dominated regime and a disorder‐dominated regime. The crystallized surface is much more stable against environmental change but lacks chemical activity in the catalytic application. While the distorted regime on the surface usually shows quite different features. A proper ratio of defects is needed to construct an active surface with minimized stability loss. The mesoscience lies in the diversity and complexity within the surface scale.

**Figure 8:**
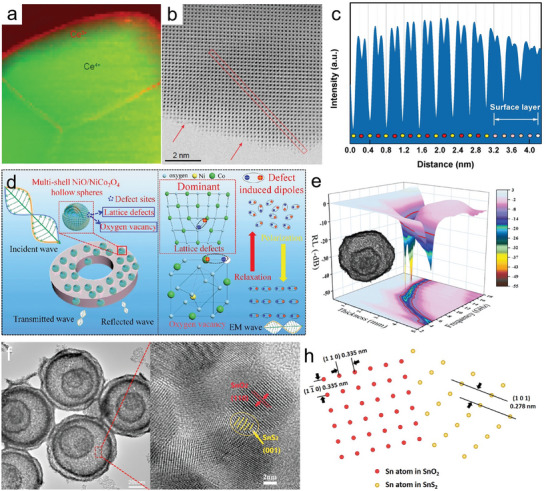
a) EELS mapping of Ce^3+^ and Ce^4+^, showing their distribution in the crystals (green: Ce^4+^ and red: Ce^3+^). b) Spherical aberration‐corrected STEM image of a subunit of 3S‐CFHoMS (the single crystal surface, with red arrows indicating the atom steps). c) Corresponding line profile of the red area in (b) with the direction from the upper left to the bottom right.^[^ ^96^ ^]^ Reproduced with permission. Copyright 2020, Oxford University Press. d) EM wave absorption mechanism of defects in NiO/NiCo_2_O_4_ HoMS. e) Reflection loss value verse frequency and corresponding 2D plots with different thicknesses for NiO/NiCo_2_O_4_ HoMS. Reproduced with permission. Copyright 2021, Wiley‐VCH. f) TEM image of SnS_2_/SnO_2_ HoMSs and corresponding enlarged HRTEM images. g) Atom model at the interface between SnS_2_ and SnO_2_ in atomic scale.^[^ ^97^ ^]^ Reproduced with permission. Copyright 2020, Wiley‐VCH.

For example, through reduction treatment, Peng et al. successfully introduced an appropriate amount of oxygen vacancies, resulting in the surface reconstruction of NiCo_2_O_4_ HoMS.^[^ ^98^ ^]^ With a proper ratio of vacancies, the stable surface configuration is formed as the result of the compromise between the perfect exposed facet and the distorted amorphous layer. Due to the optimized electronic structure, the activated surface significantly enhanced chemical activity, effectively reducing the energy barrier for the formation of intermediates. Further comprehensive studies have been conducted on surface vacancies in CeO_2_‐CeFeO_3_ HoMS. The electron energy loss spectroscopy (EELS) analysis in **Figure** **8**a reveals that Ce^3+^ is primarily concentrated at the outer surface, forming a layer ≈3.3 nm thick.^[^ ^96^ ^]^ This indicates that surface defects are located at the crystal edges and grain boundaries, while the bulk of the crystalline grain consists mainly of CeO_2_. The high‐resolution spherical aberration‐corrected scanning transmission electron microscopy (STEM) image in Figure 8b highlights the surface of a single crystal, where atomic steps are clearly visible, thus indicating the presence of missing atoms at the surface. Besides, the line profiles in Figure 8c provide evidence of oxygen vacancies on the surface. In the context of photocatalytic reactions, these vacancies serve as active sites with lower adsorption energy for reactants, leading to increased interfacial concentration and accelerated external mass transfer.

Lastly, the interaction between building blocks play a crucial role in shaping the shell structures, and this is primarily achieved through the strategic implementation of heterointerfaces during the formation of HoMS using STA.^[^ ^29^ ^]^ Besides the geometric properties, the physicochemical properties of HoMS are predominantly influenced by the interactions between building blocks.^[^ ^99^ ^]^ The combination of two or more types of building blocks leads to the formation of heterointerfaces, enabling the construction of an internal electric field that facilitates charge separation. However, excessive interfaces may introduce undesirable defects, leading to significant recombination and reduced stability, thereby hindering performance enhancement.

In the formation of HoMS with STA, the interactions between building blocks are greatly influenced by chemical compositions, which create hetero‐interfaces with different kinds of defects. For instance, Qin et al. successfully synthesized a series of HoMS composed of MCo_2_O_4_─MO (M═Mn, Fe, Ni, Cu, and Zn) through STA using templates enriched with corresponding metal precursors as EM wave absorbers.^[^ ^100^ ^]^ The diverse composition of these building blocks gives rise to distinct meso‐regimes, primarily due to variations in the crystallization process and interactions between lattices. Following the compromise in competition principle, the as‐formed building blocks exhibit different ratios of defects in the bulk or surface of the MCo_2_O_4_ spinel structure (Figure 8d). As a result, the dielectric constant of the HoMS with these distinct hetero‐interfaces with separated defect engineering directions also varies accordingly. Notably, the NiO/NiCo_2_O_4_ composite exhibits the highest number of defect sites within the spinel structure, thereby displaying the most effective electromagnetic (EM) wave absorption performance due to the highest defect‐induced polarization loss within the building blocks (Figure 8e). In this particular example, while the compromise in competition principle based on EMMS remains unchanged, it is possible to effectively regulate the electronic structure of the material by carefully manipulating the boundary conditions within the meso‐regime.

The control of the conversion of as‐synthesized building blocks through post‐treatment is highly feasible for regulating the content of heterointerfaces. Through a hydrothermal process, the implanted SnS_2_─SnO_2_ interfaces in building blocks are constructed with in situ growth of hexagonal SnS_2_ on the surface of tetragonal SnO_2_.^[^ ^97^ ^]^ The lattice distortion is confirmed by the high‐resolution TEM (HRTEM) image (Figure 8f), which demonstrates the presence of two different structural domains. The overwhelming majority of subunits exhibit interplanar spacing of both the (110) and (11—0) planes of SnO_2_, and the (110) plane of SnS_2_. The atom models (Figure 8g) display the atomic arrangement of Sn, S, and O at the interface of SnS_2_/SnO_2_, indicating the possible lattice mismatch between two distinct crystals. These lattice distortions at the interface of SnS_2_/SnO_2_ not only bring more active sites but also enhance the charge separation owing to the built‐in electric field formed by the heterogenous interface, endowing SnS_2_/SnO_2_ HoMS with enhanced catalytic performance toward photocatalytic CO_2_ reduction. Significantly, it should be noted that prolonged treatment resulting in an excess of SnS_2_/SnO_2_ heterointerfaces can have a detrimental effect on the charge transfer efficiency within SnS_2_/SnO_2_ HoMS. This is primarily due to the presence of unavoidable defects that act as recombination centers, thereby weakening the overall efficiency.

### 4.2 Mesoscience in the Shell Structures

The shell structure plays a crucial role as an intermediate boundary scale between the building blocks and HoMS. From a mathematical perspective, shells can be described as the concretization of concentration waves, as outlined in the formation mechanism of HoMS.^[^ ^60^ ^]^ Experimentally, the shell structure represents a higher‐ordered assembly that surpasses the individual building blocks, with hierarchically arranged shells serving as the foundational basis for HoMS. The physicochemical properties of these shells are heavily influenced by the intrinsic characteristics of the building blocks at the atomic scale, while also being significantly impacted by the structural features of the mesoscale HoMS.^[^ ^101^, ^102^, ^103^, ^104^ ^]^ For example, at the nanoscale, the curvature of shell structures with different geometries may not exhibit significant differences. However, at the scale of hundreds of nanometers, the morphology of shell structures can significantly impact the transmission of energy such as light waves. Additionally, the porosity of shell structures often originates from the interfaces constructed during the assembly of structural units. If the shell structure is constructed by highly uniformed polymeric or framework materials, its porosity strongly relies on the material's own periodical structure, resulting in distinct enrichment and barrier properties. In practical materials, we often encounter complex situations where multiple mechanisms are simultaneously at play, which leads to plenty of insightful case studies that highlight the importance of precise regulation of shell structures for enhancing performance in various applications. In this section, we delve into the mesoscience of the shell structures within HoMS, aiming to investigate the geometric and physicochemical characteristics that arise as a result of the compromise in competition principle.

Temporarily, the ordered multi‐shells act as physical barriers, effectively partitioning the HoMS into multiple quasi‐isolated cavities.^[^ ^1^ ^]^ In terms of spatial arrangement, these shells and cavities impose a distinct characteristic on the HoMS, causing them to undergo asynchronous changes when subjected to specific external fields such as electronic, heat, or magnetic fields.^[^ ^105^ ^]^ The manipulation of the chemical compositions of the shells can effectively harness the aforementioned characteristics of HoMS, which are primarily governed by two dominant factors.^[^ ^106^ ^]^ The A‐dominated regime refers to HoMSs with uniform compositions, while the B‐dominated regime pertains to HoMSs with diverse compositions. In the mesoregime, HoMSs possess heterogeneous shells composed of various materials, commonly known as hetero‐shells. The fabrication of hetero‐shelled HoMS has proven to be an ongoing challenge. Providentially, promising progress has been made through extensive research on STA, which offers a viable synthetic pathway by controlling the distribution and aggregation of precursors.

**Figure 9:**
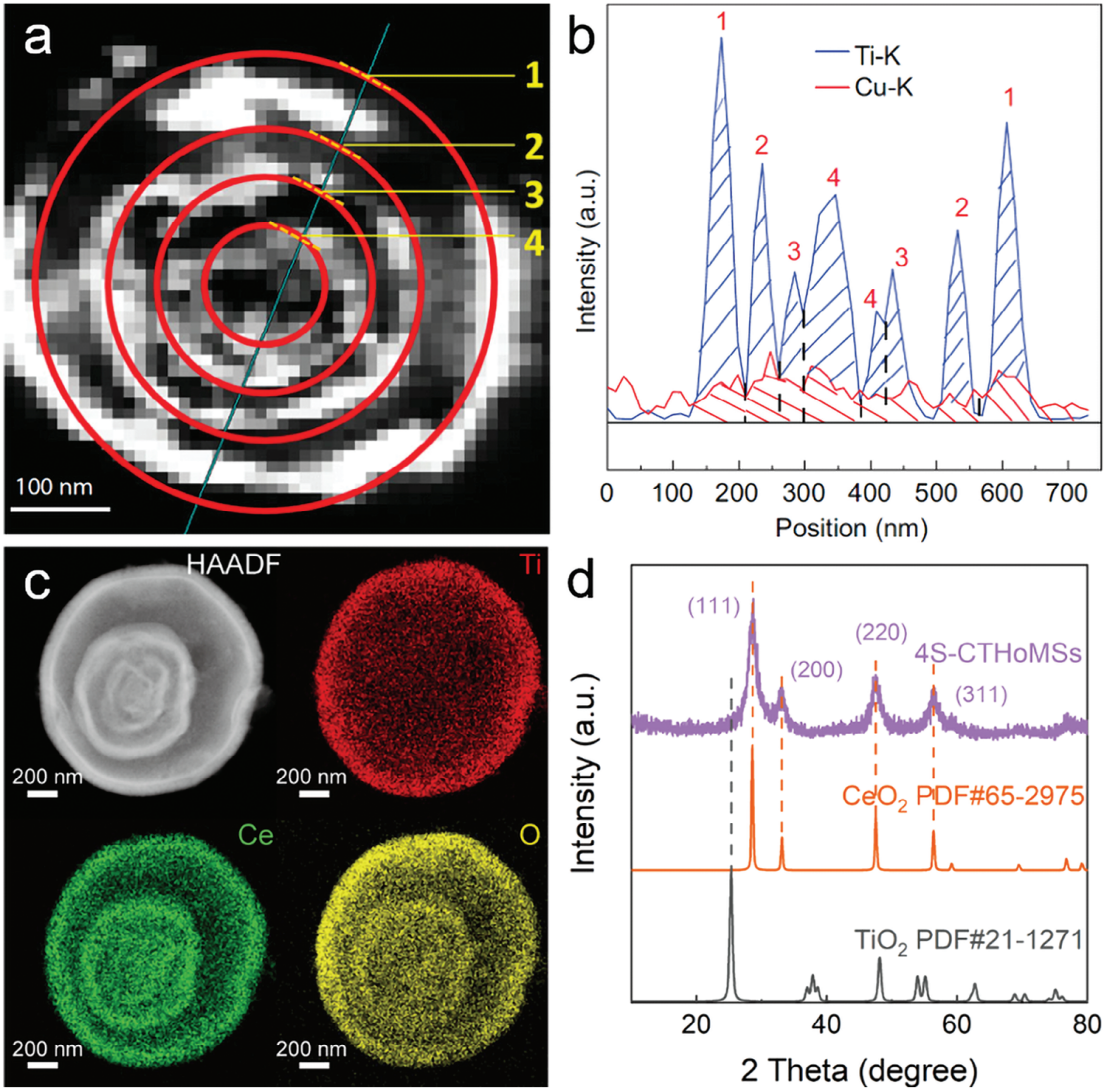
Morphological and structural characterization of HoMSs with hetero‐shells. a) Dark‐field TEM image of a slice of quadruple‐shelled TiO_2_─Cu_x_O HoMS; b) EDS line scanning along the cyan line in (a), showing the Ti/Cu ratios in different shells.^[^ ^96^ ^]^ Reproduced with permission. Copyright 2020, Oxford University Press. c) High‐angle annular darkfield scanning TEM (HAADF‐STEM) images and X‐ray energy dispersive spectral (EDS) mapping images of a single quadruple‐shelled CeO_2_@CeO_2_/A─TiO_2_ HoMS (4S‐CTHoMSs). d) XRD patterns of 4S‐CTHoMSs.^[^ ^107^ ^]^ Reproduced with permission. Copyright 2022, Wiley‐VCH.

On one hand, by effectively controlling the temporal‐spatial distribution of precursors within sequential templates, it becomes possible to achieve the desired arrangement of hetero‐shells within HoMS. For instance, Wang and his colleagues effectively utilized the spatial dimension of templates by incorporating metal ions with distinct properties (such as charge, size, and existence form) into specific positions of the sequential templates.^[^ ^96^ ^]^ Through selection of different sized [Cu(H_2_O)_4_]^2+^ and [Ti(OH)_n_(H_2_O)_6‐n_]^(4‐n)+^ ions in acid aqueous solution, they successfully controlled the adsorption of Ti and Cu precursors within the different parts of the templates. Remarkably, the distribution of Cu precursors exhibited a gradual increase from the outer to the inner regions, while the gradient of Ti precursors displayed the opposite trend (**Figure** **9**a,b). The as‐fabricated TiO_2_─Cu_x_O HoMS feature the hetero‐shells with different Ti/Cu ratios, which results in varied band structures of the shells. This outcome serves as a remarkable illustration that sheds light on the mesoscience involved in the formation of hetero‐shells. Adhering to the EMMS principle, the crystallization of the precursors faithfully preserves the spatial distribution within the templates, ultimately leading to the creation of distinct composite shells once the template is removed following a temporal procedure.

On the other hand, the accumulation behaviors and existing forms of precursors within carbonaceous templates play a crucial role in the formation of HoMSs during the STA process. Accordingly, by carefully adjusting the size of precursors and the composition of the solvent, significant variations in distribution can be achieved. Specifically, In the presence of acetone, the chemical reactions involving Ti ions and Ce ions result in the formation of precursors of different sizes. Larger‐sized Ti precursors tend to accumulate predominantly on the surface region of the templates. On the other hand, smaller‐sized Ce precursors have the ability to penetrate deeper into the core of the templates, resulting in a more extensive distribution within the templates. Simultaneously, the crystallization process of TiO_2_ is significantly influenced by the alloying effect, leading to a high affinity between TiO_2_ and Ce ions, ultimately forming amorphous TiO_2_ (A─TiO_2_) composites. As a result, the outermost shells of the synthesized HoMSs, specifically the CeO_2_@CeO_2_/A─TiO_2_ HoMS, primarily consist of A─TiO_2_/CeO_2_ composites. In contrast, the inner shells predominantly consist of CeO_2_ with an excess of Ce precursors (Figure 9c,d).^[^ ^107^ ^]^

In this section, we analyze HoMS across spatial scales, including the atomic, nanoscale, and microscale. The building blocks and shells are located at the boundaries between these scales. The physiochemical properties and regulation methods of the fabricated HoMS are influenced by both the building blocks and the shell structures. The crystal structure of the building blocks, affected by element doping, defects, and hetero‐interfaces during crystallization, greatly impacts their aggregation into shell structures, thus giving rise to distinct geometric properties and shell surfaces. The assembly of shell structures determines the sequence of shell‐cavities, which is crucial for hetero‐shelled HoMS to delineate different regions within the structure for applications requiring spatiotemporal resolution.

## 5 Applications Regarding the Energy/Mass Transfer

Thanks to breakthroughs in synthetic strategy, i.e., the STA, the precise regulation of building blocks and shell structures with different compositional and structural parameters has made it possible to customize HoMS for specific applications.^[^ ^105^, ^108^, ^109^ ^]^ After thoroughly examining the mesoscience involved in the formation and structure of HoMS, this section aims to highlight the mesoscience in energy/mass transfer within HoMS by examining several real‐life application cases that utilize the ordered shells and cavities in HoMS. Essentially, the shells and cavities within HoMS serve as barriers and plains for energy and mass transfer, giving rise to two primary mechanisms: the withholding and transport of energy/mass. By delving into the mesoscience concept within complex transdisciplinary systems, the impacts of the environment are taken into consideration. The spatially distributed periodic multi‐shell structure of HoMS, which creates a meso‐regime with changing boundary scales due to interactions with microenvironments, has brought about significant advancements in performance and nontrivial features in applications such as energy conversion, drug delivery, and catalysis. Understanding the compromise in competition phenomenon in these cases, particularly in relation to the automatic seeking of the EMMS state, holds promise for enlightening the strategy of rational design for HoMS with dynamic behaviors.

### 5.1 Mesoscience in the Energy Transfer Within HoMS

The transfer of heat, electric, and electromagnetic waves can be likened to fluid flows in the field of chemical engineering. Analogous to two‐phase flow, the energy transfer within HoMS is influenced by various factors such as transmission, reflection, and annihilation. The shells within HoMS can either fully hinder energy transfer through absorption or repulsion, while the cavities in HoMS can allow for fully unimpeded energy transfer with zero transmission loss.^[^ ^110^, ^111^ ^]^ The meso‐regime between hindered and allowed energy transfer contributes to the rich mesoscale phenomena observed in HoMS.

**Figure 10:**
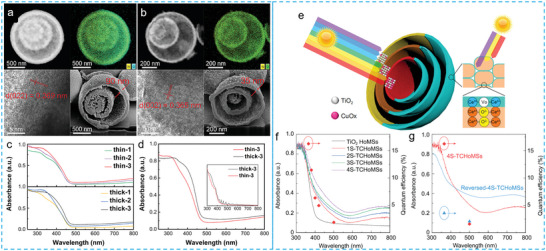
a) High‐resolution TEM (HRTEM), elemental mapping, and cross‐sectional view of thick‐3 and b) thin‐3 WO_3_ HoMS, respectively. c) UV–vis absorption spectra of thin and thick‐shelled samples. d) Comparison of the light‐harvesting ability between thin‐3 and thick‐3 WO_3_ HoMS, and inset is the calculated absorption results obtained through the finite element method.^[^ ^78^ ^]^ Reproduced with permission. Copyright 2021, Royal Society of Chemistry. e) Illustration of two designed heterogeneous HoMS for efficient sequential harvesting of solar light. f) UV–vis absorption curves of TCHoMS with different shell numbers and apparent quantum efficiency (red diamonds) of 4S‐TCHoMS at different wavelengths. g) UV–vis absorption curves of 4S‐TCHoMS, TiO_2_‐Cu_x_O nanoparticles, crushed 4S‐TCHoMS and TiO_2_ HoMS.^[^ ^96^ ^]^ Reproduced with permission. Copyright 2020, Oxford University Press.

Taking the transfer of light within HoMS as an example, the shells and cavities play distinct roles.^[^ ^112^ ^]^ Although the absorbance of incident light is mainly achieved by shells, the geometry properties of cavities strongly affect the intensity of Mie scattering within HoMS.^[^ ^113^ ^]^ Therefore, by regulating the structure of HoMS, it is possible to harmonize light absorbance and scattering, thereby increasing the overall light absorption ability.^[^ ^111^ ^]^ Building upon this perspective, Zhang et al. conducted a comprehensive study on the influence of shell thickness on the light harvesting capability of WO_3_ HoMS (**Figure** **10**a,b). It was found that thin‐shelled WO_3_ HoMS with a shell thickness of 35 nm exhibited higher UV light adsorption below 350 nm, thanks to their enhanced scattering ability. On the other hand, thick‐shelled WO_3_ HoMS with a shell thickness of 90 nm demonstrated superior light‐harvesting performance for light wavelengths above 350 nm (Figure 10c,d). The variation in shell thickness effectively altered the dominance of shells and cavities, resulting in different light absorption performances for incident light with varied wavelengths.^[^ ^78^ ^]^

The compromise between the dominance of shells and cavities in the defined meso‐regime has provided valuable insights into a novel strategy for enhancing light harvesting efficiency, which involves the absorption of different wavelength light using distinct shells within HoMS, thereby maximizing the overall light harvesting capacity of HoMS. In a remarkable coincidence, the antenna systems of cyanobacteria and chloroplasts in plant cells possess a spatial arrangement that optimizes the efficient capture of light.^[^ ^114^ ^]^ The pigments sensitive to short wavelength light are strategically positioned in the outer regions, gradually transitioning to pigments responsive to longer wavelength light as they approach the center.^[^ ^115^ ^]^ Inspired by the ingenious design, TiO_2_Cu_x_O HoMS with heterogeneous shells with semiconductors with different band gaps are fabricated (Figure 10e).^[^ ^96^ ^]^ Specifically, the gradual increase in Cu/Ti ratio on the shells, from the outside‐in, allows for the sequential absorption of short‐wavelength light, which is weakly penetrable, by the outer shell, and long‐wavelength light, which is strongly penetrable, by the inner shells. This process effectively widens the spectrum of light absorption and enhances t‐─he overall ability to absorb light (Figure 10f). The improved light‐harvesting ability can be further observed by comparing the UV–vis spectra of TCHoMS with those of TiO_2_─Cu_x_O nanoparticles (TCNPs), TiO_2_ HoMS, and the corresponding crushed 4S HoMS (Figure 10g). The disruption of the highly ordered structures significantly affected light scattering and resulted in reduced light absorption across a wide range of wavelengths. Interestingly, despite the increased exposure of Cu_x_O content in the inner shells after crushing the HoMS, there was still a slight decrease in light absorption in the visible light region. Furthermore, 4S‐TCHoMS with a reversed composition of TiO_2_ and Cu_x_O were synthesized to highlight the significance of the light‐harvesting sequence. Due to the higher Cu_x_O content in the outer shells, the reversed 4S‐TCHoMS exhibited an improvement in visible light absorption. However, the absorption of UV light was significantly hindered. In this condition, there was an increased scattering of short‐wavelength light, which dominated the light transfer process and led to a loss in absorption in this high‐energy region.

Notably, in these hetero‐structured light absorbers, the meso‐regime dominated by the compromise and competition between absorbance and scattering undergoes dynamic changes. The spatial adaptation of shell compositions enables the efficient capture of incident light across a wide range of wavelengths, facilitating the sequential harvesting of solar light with a broad spectrum. Definitely, this strategy extends beyond the preparation of hierarchical light conversion materials structure and can be readily applied to the development of electromagnetic wave absorption materials.

#### 5.1.1 Mesoscience in the Mass Transfer Within HoMS

**Figure 11:**
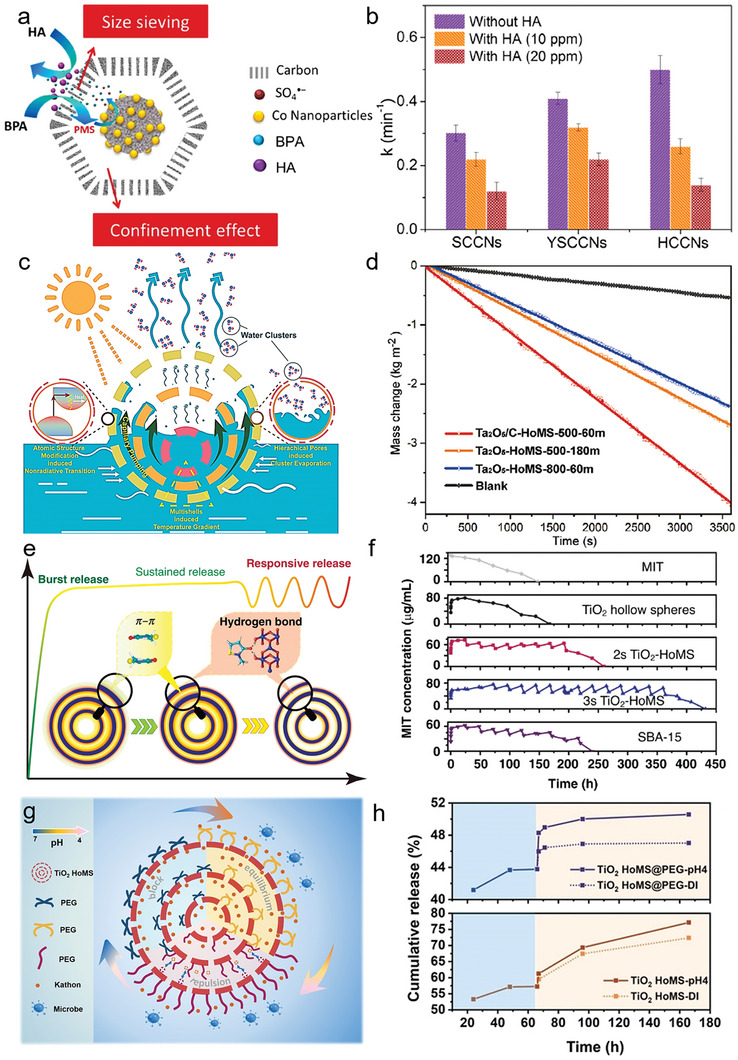
a) Schematic illustration of the synergistic mechanisms of size sieving and confinement effect in Co/C HoMS nanoreactors. b) the comparison of rate constant k for three samples.^[^ ^122^ ^]^ Reproduced with permission. Copyright 2020, American Chemical Society. c) Schematic of highly efficient solar‐to‐vapor generation via amorphous Ta_2_O_5_/C HoMS. The water transport within the system is modified, serving two merits. Firstly, it facilitates rapid water transportation via the capillary pumping effect. Secondly, it alters the manner in which water evaporates through the nanopores, causing it to evaporate in the form of small clusters. d) The mass loss of water with the corresponding evaporation rate of various HoMS samples under 1 sun illumination.^[^ ^91^ ^]^ Reproduced with permission. Copyright 2021, Wiley‐VCH. e) The schematic illustration depicts the spatially dependent drug release mechanism of TiO_2_ HoMS. The green part represents the burst release of drug molecules that are adsorbed on the outer surface of the shells; the yellow part represents the sustained release of drug molecules residing in the cavities of the HoMS; and the red part represents the responsive release of drug molecules that form hydrogen bonds with the TiO_2_ HoMS carriers. f) Bacterial‐responsive release profiles of corresponding samples.^[^ ^126^ ^]^ Reproduced with permission. Copyright 2020, Springer Publishing Group. g) Schematic illustration of the pH‐responsive antimicrobial release mechanism of TiO_2_ HoMS@PEG.^[^ ^127^ ^]^ h) pH‐responsive release performance of 3s‐TiO_2_ HoMS and 3s‐TiO_2_ HoMS@PEG.^[^ ^127^ ^]^ Reproduced with permission. Copyright 2022, Wiley‐VCH.

When considering the mass transfer within HoMS, the shells, and cavities assume distinct roles that give rise to two extremes: the confinement effect induced by cavities, which significantly obstructs the release of molecules, and the free transport behavior facilitated by porous shells, which effectively enhances the transfer of molecules.^[^ ^116^ ^]^ The EMMS principle within these two extremes endows HoMS with unique mass transfer features.^[^ ^117^ ^]^ Obviously, the structural parameters of the shells would modulate the competition between two extremes, ultimately determining the efficiency of mass transfer through a compromise. By simplifying HoMS as a rigid geometric structure and disregarding the interactions between molecules and shells, the mass transfer rate through the shells can be indirectly estimated by considering the surface reaction rate (*r*) using the Weisz–Prater modulus (φ’), which is described as:

(3) $$r=\frac{\varphi^{\prime }D\varepsilon c}{d_{L}^{2}\tau }$$

where *D* is the diffusion coefficient, *ε* is the porosity of the shells, *c* is the concentration at the interface, *d*_L_ is the characteristic dimension, and *τ* is the tortuosity of the shells. Since *D*, φ’, *d*_L_ and *τ* remain constant for specific HoMS, the varying of *ε* and *c* are crucial for achieving boosted internal mass transfer.^[^ ^118^ ^]^ The porosity of shells has a great influence on the diffusion of molecules with different sizes and mechanical stability.^[^ ^119^ ^]^ By controlling the pore size, the passage and blocking of specific molecules can be regulated, in which the compromise in competition within the meso‐regime would endow HoMS with selective permeability.^[^ ^120^, ^121^ ^]^ For instance, starting from ZIF‐67, the double‐shelled Co/C HoMS‐based nanoreactors (YSCCNs) are fabricated through pyrolysis of controllably acid‐etched ZIF‐67 (**Figure** **11**a).^[^ ^122^ ^]^ These nanoreactors inherit the pore size from the ZIF‐67 templates, leading to competition among molecules to migrate into the Co/C HoMS. As a result, bisphenol A (BPA) can be selectively harvested into the nanoreactors, while larger molecules are excluded. In comparison to the degradation performance of solid and hollow Co/C nanoparticles (SCCNs and HCCNs) toward BPA, YSCCNs demonstrate superior degradation efficiency in the presence of humic acid (HA) which is often encountered in practical applications, as shown in Figure 11b. The nanoreactor's ability to selectively sieve specific molecules enables it to exhibit responsive behaviors, effectively balancing competition and compromise through two dominant transfer mechanisms. This size‐sieving capability demonstrates the significance of comprehending and manipulating the mesoscale in mass transfer when utilizing HoMS for targeted contaminant removal from complex solution systems.^[^ ^123^ ^]^ Moreover, under extreme conditions, the interactions among the transported molecules are influenced by the pore structure. This altered pore structure forces the molecules to aggregate in a way that accommodates the modified structure, leading to a new compromise in the meso‐regime.^[^ ^124^ ^]^ For example, Chen et al. have developed a method to fabricate amorphous shells on Ta_2_O_5_/C HoMS, allowing us to optimize the pore size on the shell and enhance the capillary force (Figure 11c).^[^ ^91^ ^]^ When applying the Ta_2_O_5_/C HoMS with a pore distribution ranging from 1 to 2 nm in the photothermal conversion process for water evaporation, they observed that water molecules tend to escape from the nanopores of HoMS as small clusters rather than individual molecules.^[^ ^125^ ^]^ A faster water transport is then realized through optimized actual water evaporation enthalpy, resulting in a faster water evaporation rate under illumination. Besides, the diminished size of water clusters leads to a significant decrease in salt concentration as a result of the decreased interactions between water molecules and ions. This, in conjunction with the presence of amorphous domains in the Ta_2_O_5_/C shells, allows the Ta_2_O_5_/C HoMS based solar water evaporation system to achieve an impressive water evaporation speed of 4.02 kg m^−2^ h^−1^ (Figure 11d). Not only does this system demonstrate exceptional desalination and purification capabilities, but it also validates the advantageous impact of the modulated pore structures on the photo‐thermal water generation performance.

In another attempt, TiO_2_ HoMS are employed as drug carriers for loading the antibacterial agent methylisothiazolinone (MIT) (Figure 11e).^[^ ^126^ ^]^ Within the meso‐regime, this unique system enables the achievement of a smart stimuli‐responsive release, bridging the gap between burst‐release and sustained release. Specifically, the HoMS‐based drug carriers exhibit three distinct release stages. The first stage involves a burst release, attributed to the desorption of physically adsorbed MIT molecules. The second stage is characterized by sustained release, facilitated by π–π interactions among the MIT molecules. Finally, the stimulus‐responsive release occurs as a result of the disruption of hydrogen bonding between stored MIT molecules and the surface of TiO_2_ shells.^[^ ^128^ ^]^ The presence of these three distinct stages enables a smart detachment response to external stimuli, making TiO_2_ HoMS highly adaptable for various treatment scenarios. As depicted in Figure 11f, this stimulus‐responsive release, i.e., the third stage of sequential release, is a unique feature of the HoMS system by creating a dynamic compromise within the competitive micro‐environment, allowing for the controlled release of drug molecules. To investigate the responsive release performance, bacteria were introduced to the solution after stabilizing the concentration of MIT across various samples including MIT, MIT─TiO_2_ hollow spheres, MIT─2s─TiO_2_–HoMS, MIT‐3s‐TiO_2_–HoMS, and SBA‐15. It was observed that 2s‐ and 3s‐ TiO_2_–HoMS, as well as SBA‐15, all exhibited responsive release behavior. Following the rapid decrease in MIT concentration, equilibrium was gradually restored. Notably, 3s‐ TiO_2_–HoMS demonstrated the most efficient recycling performance among all samples, maintaining the concentration above the minimum inhibitory concentration even after 14 rounds of bacteria addition. In the abovementioned case, the structural parameter is fixed for TiO_2_–HoMS during the whole release process to remain stable, where the micro‐environment of the system dominates the rapid or restricted transfer of MIT molecules.

Furthermore, gaining a comprehensive understanding of the interaction between molecules and HoMS is crucial for comprehending its distinctive mass transfer behavior, thereby opening up new possibilities for tailoring the mass transfer efficiency to suit various application scenarios. Accordingly, Zhao et al. have successfully modified the surface of TiO_2_ HoMS by incorporating polyethylene glycol (PEG) as a gated regulator, which is fully covered on the surface of shells (Figure 11g).^[^ ^127^ ^]^ This modification enables the interaction between the PEG‐functionalized shells and drug molecules to be controlled by the pH of the environment, thus providing the HoMS with a pH‐responsive switch and rate‐regulator capability. Specifically, the release of Kathon drugs is impeded in a neutral environment with a pH of 7, while a significant increase in release efficiency is observed when the pH is decreased to 4 (Figure 11h). This change in the aqueous environment greatly affects the boundary of the meso‐regime of the mass transfer behavior within the HoMS, which reconstructs the balance. Moreover, the dynamic interactions resulting from the variation in the microenvironment reflect the oscillation of the compromise in competition between two different dominant mechanisms, which further demonstrates the impact of the changing meso‐regime on the mass transfer properties of the HoMS. As a result, achieving dynamic mass transfer with a single HoMS becomes feasible by finely regulating the meso‐regime through the precise design of the interactions between the drug molecules, HoMS, and the microenvironments. This opens up a new pathway for the development of novel long‐term antimicrobial materials with smart stimuli‐responsive behaviors.^[^ ^129^ ^]^

In this section, we unveil the mesoscience behind the mass/energy transfer behavior of HoMS, revealing the distinct roles played by shells and cavities, which give rise to a unique temporal‐spatial ordered transport mechanism. When viewed from a larger spatial scale, the internal structure of HoMS forms a mesoscale, where the combined mechanisms of shell structure and cavity sequentially influence the obstruction and transmission of matter/energy, thus resulting in transport characteristics that are distinct from solid particles and simple hollow structures. Additionally, when zooming in on a single HoMS, the shell structure acts as another boundary scale, affecting mass/energy transport through factors such as thickness, porosity, crystal structure, and interfacial interactions. The response of HoMS to factors such as incident light and pH environment is a result of the interplay between these two mechanisms. To further develop the unique applications of HoMS in material/energy transfer processes, precise utilization of the spatial depth of HoMS is required, which is crucial for the temporal activation of different cavities and shells.

## 6 Conclusion and Perspective

**Figure 12:**
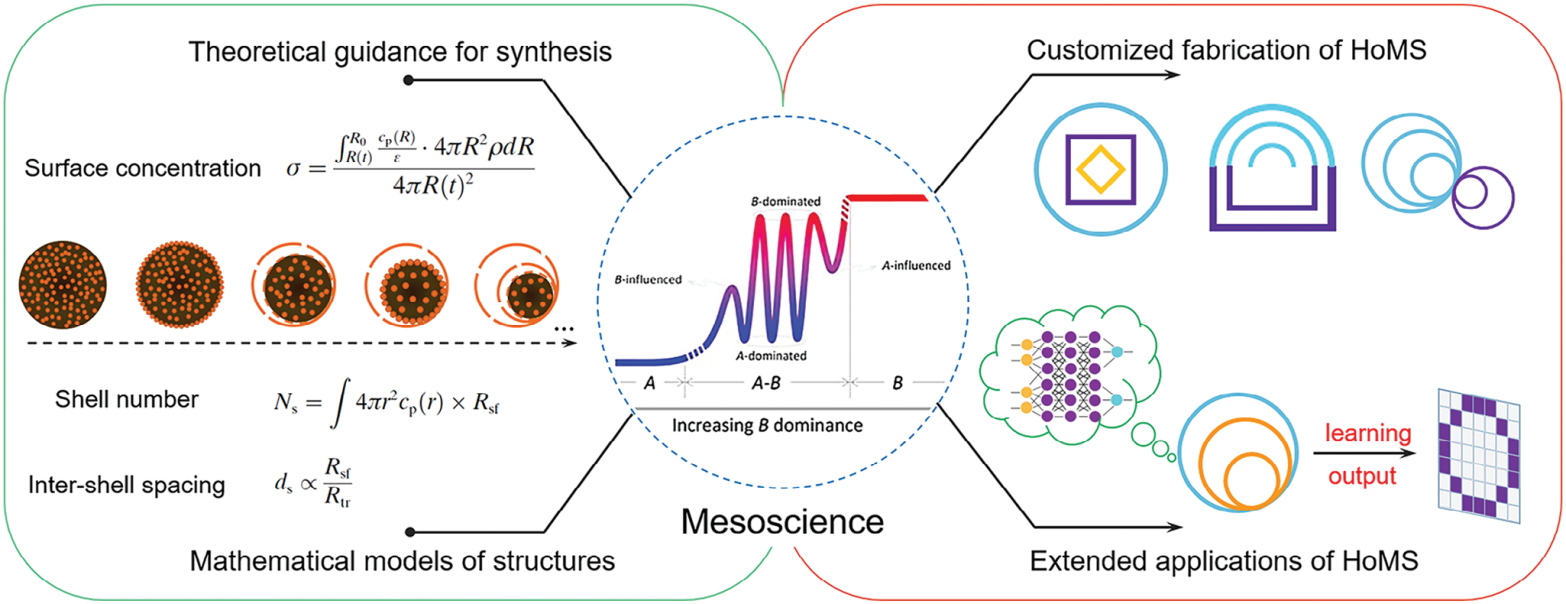
New orientations for future development of HoMS in the perspective of mesoscience.

In this review, from the vista of mesoscience scope, we conducted a comprehensive analysis of the mesoscale issues involved in the formation process of HoMS, the structural parameters of HoMS, and performance in energy/mass transfer, where the compromise in competition or EMMS principle roles. Firstly, the formation process of HoMS exhibits a prominent mesoscale phenomenon, primarily characterized by STA. Through experimental case studies, numerical simulations have revealed the delicate balance between *R*_sf_ and *R*_tr_, shedding light on the compromise between the dominant mechanisms of oxygen diffusion and template reaction. Moreover, the precise regulation of shell parameters is directed through a meticulous control of the competition between these two mechanisms. Secondly, the building blocks and shell structures that constitute HoMS represent two distinct spatial boundaries, symbolizing the individual and the system. The intimate interaction between the crystal structure of the building blocks and the aggregation configuration of the shells significantly impacts the electronic structure, geometry, and physicochemical properties of HoMS. The results also highlight the dynamically changing boundary scales due to the changed dominance, which reveals the competition between the individual and the system, in accordance with the principles of EMMS. Finally, in various application scenarios of HoMS, the shells, and cavities play distinct characters in energy and mass transfer, leading to the emergence of two dominant mechanisms in the meso‐regime: the withholding and transport of energy/mass. Taking into account the spatial and temporal factors, the strategically distributing shell structures realize the sequential response to the input energy and balance the retention and transportation of molecules for controlled release. By establishing the crucial connection between process and appearance, mesoscience offers a fresh perspective for comprehending the intricate structure‐activity relationship within HoMS, which enhances our ability to unravel the underlying mechanisms governing these complex material systems **Figure** **12**.

Despite the new findings from the view of mesoscience, challenges still remain in precisely predicting the compromising results within the meso‐regime, as the presence of multiple dominant mechanisms at play would lead to unpredicted compromised outcomes. Moreover, the inherent complexity of the practical meso‐regime poses a significant challenge in establishing precise mathematical expressions to resolve the intricate interactions between dominant mechanisms or boundary scales with temporal‐spatial resolution. Fortunately, as the awareness of mesoscience in complex material systems continues to grow, there is great potential for future advancements in both mesoscience in materials science and HoMS. In light of the aforementioned challenges, we propose the following outlook:

First, after gaining a deep understanding of the physical essence behind the formation process of HoMS, the theoretical guidance makes it possible to manipulate the synthesis of HoMS with highly intricate compositions and morphologies by applying external stimuli to specifically designed sequential templates. This can effectively induce concentration waves within a gentle and controlled environment for realizing the green and large‐scale synthesis of HoMS. Besides, for developing the understanding of mesoscience in HoMS, the variation of boundary scales should be taken into consideration, especially in meso‐regimes closely related to chemical reactions or obvious microenvironment variations. The changes in systems in both time and space may call for distinct applicative dominance. For example, Newtonian mechanics dominates the aggregation of building blocks to form shells, while the interfaces between building blocks are ruled by quantum mechanics. New mesoscience issues may arouse from the changing of dominant mechanisms.
Second, within the theoretical framework of the “compromise in competition” mechanism in mesoscience, a robust mathematical model will be established to comprehensively study the structural and performance changes of HoMS resulting from alterations in the boundary scales of building blocks and shell structures. Moreover, it is essential to leverage the advancements in in situ techniques to accurately describe the structural properties of HoMS and continuously optimize the model. These evolving experimental techniques have the potential to provide more quantitative evidence regarding the immediate dominant mechanism following A‐B compromising. By utilizing in‐situ electron microscope techniques in an atmospheric environment to detect the crystal structure and shell composition during the shell formation process, the reaction of precursors can be easily taken into consideration. This comprehensive approach, combined with the aforementioned analysis of interactions, is expected to yield valuable insights for machine learning predictions of HoMS synthesis and structural modulation from a mesoscale perspective.
Lastly, in conjunction with the advancements in mesoscience related to the formation and structure of HoMS, it is evident that for maximizing the performance of HoMS, the meso‐regime comprising of shells and cavities indicates the need for dynamic changes in shell structures in response to variations in the microenvironment. Utilizing the mathematical model of HoMS, it is anticipated that the mass/energy transfer within HoMS can be accurately predicted. Furthermore, the dynamic adaptation of HoMS can be theoretically illustrated by taking into account the stimuli‐responsive interactions between building blocks and shell structures. These collective efforts lay a solid foundation for exploring new applications of HoMS, including the development of HoMS‐based devices for artificial neural networks used in supervised learning.

Overall, the comprehensive analysis of mesoscale issues in HoMS would shatter many boundaries in the synthesis, characterization, and application of HoMS. Hopefully, new research interest regarding the complex meso‐regimes would spark novel concepts and irreplaceable applications of these multifunctional mesoscale HoMS systems.

## Conflict of Interest

The authors declare no conflict of interest.
